# Major changes in indoor air-related symptoms, health worry, and views between 2018 and 2022 in Finland

**DOI:** 10.1186/s12889-025-24224-8

**Published:** 2025-10-10

**Authors:** Einar Eidstø, Sanna Selinheimo, Kati Huttunen, Vuokko Härmä, Tiina Laatikainen, Pekka Jousilahti, Anniina Salmela, Juha Pekkanen

**Affiliations:** 1 Department of Public Health, Faculty of Medicine, University of Helsinki, Helsinki, Finland; 2 Finnish Institute of Occupational Health, Helsinki, Finland; 3 Department of Public Health, Finnish Institute for Health and Welfare, Kuopio, Finland; 4 Communications and Influencing Unit, Finnish Institute for Health and Welfare, Helsinki, Finland; 5 Institute of Public Health and Clinical Nutrition, University of Eastern Finland, Kuopio, Finland

**Keywords:** Indoor air, Moisture damage, Health worry, Risk perception

## Abstract

**Background:**

Indoor air-related symptoms and health-worry are common in Finland, even though the levels of indoor air pollutants are mostly lower than elsewhere in Europe. To address these problems, the Finnish Indoor Air and Health Programme 2018–2028 was initiated. Changes in the population were monitored with repeated surveys.

**Methods:**

Questionnaire surveys were conducted in two independent random samples of Finnish people aged 25–64 in 2018 and 2022, with 1797 and 1616 responses, respectively. Changes in the prevalence of symptoms and other factors were investigated using multivariate logistic regression. Findings on the prevalence of symptoms were replicated in two additional major national surveys.

**Results:**

Several significant changes were observed. In 2018, 45.8% reported worry related to the health effects of indoor air, compared with 26.8% in 2022. Indoor air-related symptoms at work decreased from 21.3 to 16.2%, and more respondents deemed the indoor air at their workplace as good. Moderate to severe symptoms decreased from 10 to 8%. Smaller improvements were observed in knowledge about the health effects of indoor air. Changes tended to be stronger among women and among those with an academic degree, less trust in social media, and less severe symptoms.

**Conclusions:**

Four years after the Finnish Indoor Air and Health Programme was initiated, several improvements occurred in the population in self-reported symptoms, perceived indoor air quality, health worry, and knowledge. Although it is impossible to disentangle the different causes for these changes by repeated surveys, it is likely that the programme has contributed to the positive developments observed.

**Supplementary Information:**

The online version contains supplementary material available at 10.1186/s12889-025-24224-8.

## Background

Besides the adverse impacts of indoor air-related symptoms on the health and well-being of individuals [1–4], poor indoor air quality also imposes a substantial financial burden on society, e.g. through decreased productivity [5] and renovation costs [6]. During the past three decades in Finland, a lot of effort has been put into improving indoor air quality, with a particular focus on decreasing moisture damage and mould in buildings [7]. Although the levels of most indoor air pollutants are lower in Finland than elsewhere in Europe [7–9], indoor air- or building-related symptoms [1, 3] and indoor air-related health worry [10, 11] are nevertheless common. Media coverage regarding indoor air-related issues has been very frequent in Finland [12], and the opinions of the public on indoor air and its health effects often differ from current scientific knowledge [13]. Therefore, new evidence-based methods and approaches were needed to address indoor air-related symptom reports, health worries, and misconceptions.

In spring 2017, the Finnish Institute for Health and Welfare initiated the preparation of the Finnish Indoor Air and Health Programme 2018–2028 [7] together with collaborators, such as the Finnish Institute of Occupational Health, and stakeholders, such as medical professionals and people with indoor air-related symptoms. The programme drew on the experiences of past successful population-based programmes in Finland: The Finnish Asthma Programme and the Finnish Allergy Programme [14, 15]. The programme was designed using the logical framework approach method in a total of eight workshops and other planning meetings. In addition, extensive stakeholder hearings were held, and the recent scientific literature on relevant topics was reviewed. The programme collaborates with and is partly financed by a wider government programme focusing on promoting well-being on public premises [7].

The primary objective of the Indoor Air and Health Programme is to reduce indoor environment-related hazards to health and well-being in Finland. Already during the planning phase of the programme, it was recognised that simply reducing indoor air pollution is not enough, but that other measures would also be needed [7]. Therefore, the programme was designed to consist of four areas: informing the general public of the health effects of indoor environments (area 1), improving the management of indoor air-linked problems in buildings (area 2), developing the treatment and support of people with indoor air-related symptoms and illnesses such as to improve their functional and work capacity (area 3), and education and training of actors, e.g. property owners, building managers, and medical professionals, in indoor environment-related issues (area 4) [7].

The fulfilment of the programme’s objectives was planned to be monitored using predefined indicators halfway through the programme in 2023 and at the end of the programme in 2028. For the main objective and areas 1 and 2, progress has been monitored through surveys, while for areas 3 and 4 progress has been monitored through outcome indicators, i.e. assessing the implementation of planned actions [7]. The actions of the programme are described at the beginning of Methods and further in Additional file 1.

This study contributes to the intermediate evaluation of the programme using mainly data from National Surveys on Indoor Air, which were first carried out at the beginning of the programme in 2018 and again in 2022. We are not aware of any similar previous assessments in the field of indoor air. This report focuses on the changes in the prevalence of the primary indicators of the programme, i.e. indoor air-related symptoms and perceived indoor air quality. In addition to National Surveys on Indoor Air, data from two other population-based surveys are used. We also report results on risk perceptions, on the satisfaction of the public with the actions of municipalities relating to indoor air, and on knowledge about indoor air-related health effects, which relate to areas 1 and 2 of the programme. We also explore differences in the observed changes between subgroups of the population.

## Methods

### Intervention

To achieve its goals, the Finnish Indoor Air and Health programme performed a large number of actions [16]. These included disseminating information through press releases, online materials banks, updated websites, blogs, interviews, lectures, and discussions with stakeholders. The programme highlighted, among other things, that factors beyond indoor air pollutants also influence indoor air-related symptom reporting, making symptoms an uncertain way of assessing indoor air quality. The management of indoor air problems involved refining management processes, clarifying the responsibilities and roles of different actors, and enhancing open communication and the assessment of exposures and health effects related to indoor environments. In order to facilitate this work, extensive reviews were conducted on indoor factors and their health effects, including the promotion of well-being. New guidelines have also been written for residents regarding housing health and cleaning after moisture damage repairs. Enhancements in health and social care and support for individuals with indoor air-related symptoms included supporting the establishment of rehabilitation clinics and creating web-based materials on e.g. multiple factors affecting indoor-air related symptoms and treatment options. Regarding the education of professionals, materials have been published on online platforms accessible to all health care professionals. In addition, training and materials have been provided for workplaces, indoor environment experts, occupational health specialists as well as for employees. A more comprehensive description of the actions of the programme is included in Additional file 1.

### Study populations

Our data is derived from two questionnaire-based surveys, National Survey on Indoor Air (NSI) 2018 and 2022. In the 2018 and 2022 surveys, a postal questionnaire was sent to a random sample of, respectively, 4997 and 5000 Finnish speakers (25–64 years old) in Finland, excluding Åland, the autonomous Swedish-speaking part of Finland. The questionnaire with all response categories is shown in Additional file 2. In 2018 and 2022, 1797 (36%) and 1616 (32%) subjects, respectively, responded either to the postal or the electronic questionnaire. The data collections were carried out during the winters of 2018–2019 and 2022–2023, spanning from October to February.

Part of the results from the NSI were compared with two major national surveys, which collected data using identical questions on indoor air-related symptoms and visits to a physician, the FinHealth survey (FH) in the spring of 2017 [17] and the Healthy Finland Survey (HFS) in the spring of 2023 [18]. These surveys monitored the health and well-being, and use of social and health services of adults living in Finland in nationally representative samples of the Finnish population. Randomly selected subjects (*n* = 10 247) aged over 18 years living in 50 locations in Finland were invited to participate in the FH survey and 58.1% responded. Finns (*n* = 9862) aged over 20 years living in the same 50 locations as in FH were invited to participate in the HFS survey and 51.3% responded.

### Outcomes and variable coding

#### Indoor air-related symptoms

Respondents were asked, *“Have you ever had any symptoms from indoor air at home?”*. Three response options were provided (*No symptoms*; *yes*,* during the past 12 months*; *yes*,* over 12 months ago*). The same question was asked about symptoms from indoor air at work. Those who reported symptoms during the past 12 months at home or at work or both were considered symptomatic and those who reported no symptoms or had had symptoms only over 12 months ago were considered non-symptomatic. The respondents were similarly asked whether they had been examined or treated by a doctor due to symptoms suspected to be mainly caused by poor indoor air during the past 12 months. These answers were also dichotomised (*Yes*,* during the past 12 months* vs. *No*,* not during the past 12 months*).

The above questions were provided both in NSI 2018 and 2022, and also in FH 2017 and HFS 2023. The rest of the questions described here were provided only in the NSI.

NSI in 2018 and 2022 also asked whether the respondent had been on sick leave due to poor indoor air during the past 12 months. Also, due to the COVID-19-pandemic and the subsequent changing telecommuting habits, a question was added to the 2022 survey: *“During a typical work week*,* how many hours per week do you spend indoors at work or school? Think primarily about the previous 4 weeks. Remote work or work done outside of the building doesn’t count.”* Answers were given in hours (h) with whole numbers and dichotomised for the analysis (*30 h or less* vs. *over 30 h*).

Those individuals who reported symptoms either at home or at work were further asked, *“How severe symptoms have you experienced from indoor air at home or at work in the past 12 months?”* A four-point scale was provided (*mild*, *moderate*, *severe*, *very severe*). Due to low numbers, the last two categories were combined to form 4 groups: *no symptoms*, *mild symptoms*, *moderate symptoms*, *severe symptoms*. The participants were asked to rate the indoor air quality at their home and their workplace separately on a five-point scale from *‘Very bad’* to *‘Very good’*. In the analysis, indoor air quality was dichotomized (*Very good or good* vs. *average*,* bad*,* or very bad*). ‘Average’ was grouped with ‘bad or very bad’ due to our interest in the group ‘good or very good.’ In this and other Likert-style questions, the regrouping was made such that each group had enough people for statistical analysis. The participants were likewise asked about their sensitivity to experience symptoms from indoor air compared to other people. There were four options from *‘No’* to *‘Yes*,* very much more likely’*. In the analysis, sensitivity to experience symptoms was dichotomized (*Much or very much more likely* vs. *No or a little more likely*).

Respondents were provided with a list of possible health hazards and asked, *“And how much risk to human health in general do you consider these factors to be in Finland?”* Answers included a five-point scale from *‘No risk at all’* to *‘Very high risk’* and the option *‘I cannot say.’* In the analysis, risk perceptions were dichotomized (*Large or very large risk* vs. *moderate to no risk/cannot say*). ‘I cannot say’ was grouped with ‘moderate to no risk’ due to our interest in the group ‘large or very large risk.’ Indoor air-related health worry was measured with the following statement: *“I’m very worried about the health effects of indoor air in Finland.”* The participants were asked to rate this, and other statements related to indoor air problems on a five-point scale from *‘Fully disagree’* to *‘Fully agree’*. In the analysis, the answers were dichotomized (*Fully agree or agree* vs. *Fully disagree*,* disagree*,* or neither*). Respondents were further asked, *“How satisfied are you with the actions of your municipality in indoor air-related matters?”* Five response options were provided from *‘Very dissatisfied’* to *‘Very satisfied.’* The same question was asked about four specific matters related to indoor air.

Knowledge of the health effects of indoor air was inquired: *“Mere concern about indoor air quality can effect symptoms similar to indoor air symptoms”* and other such statements. There were six response options (*fully disagree* to *fully agree* and *cannot say*). In the main analysis, the responses were recategorized into three groups (*disagree*, *neither/cannot say*, *agree*), but in the subgroup analyses, they are further dichotomized (*Disagree* vs. *other*).

### Covariates and their coding

Respondents were inquired whether they were living in *‘owner-occupied housing’*, *‘a rented apartment’*, *‘a housing cooperative or similar’*, or *‘somewhere else’*. In the analysis, this was dichotomized (*owner-occupied housing* vs. *other*). Four options were provided to describe place of residence: *inner city*, *suburbs*, *countryside population centre*, *dispersed settlement*. Place of residence was dichotomized (*urban area* vs. *rural area*). Form of housing also had four options (*single-family home*, *semidetached house or terraced house*, *apartment building*, and *something else*). In the analysis, form of housing was dichotomized (*single-family home* vs. *other*). Marital status was dichotomized into *‘married’* and *‘not married’*, employment into *‘(self-)employed’* and *‘other’*, and education into *‘academic degree’* and *‘no academic degree’*. Those employed full-time or part-time were not differentiated. Academic degree refers here to any tertiary degree. Participants were asked, *“Do you currently smoke?”* with 7 options from *‘not at all’* to *‘daily*,* over 15 cigarettes a day’*. In the analysis, smoking was dichotomized into those who never smoke and those who at least sometimes smoke. Trust in specific authorities (e.g. *media*, *social media*, and *state and municipal officials*) on indoor air matters was asked on a five-point scale, which was dichotomized (*trust to full trust* vs. *moderate trust to none*). Due to data confidentiality issues, age was available only in 5-year categories, and it was treated as a continuous variable in the analyses.

### Statistical analysis

**Fig. 1:**
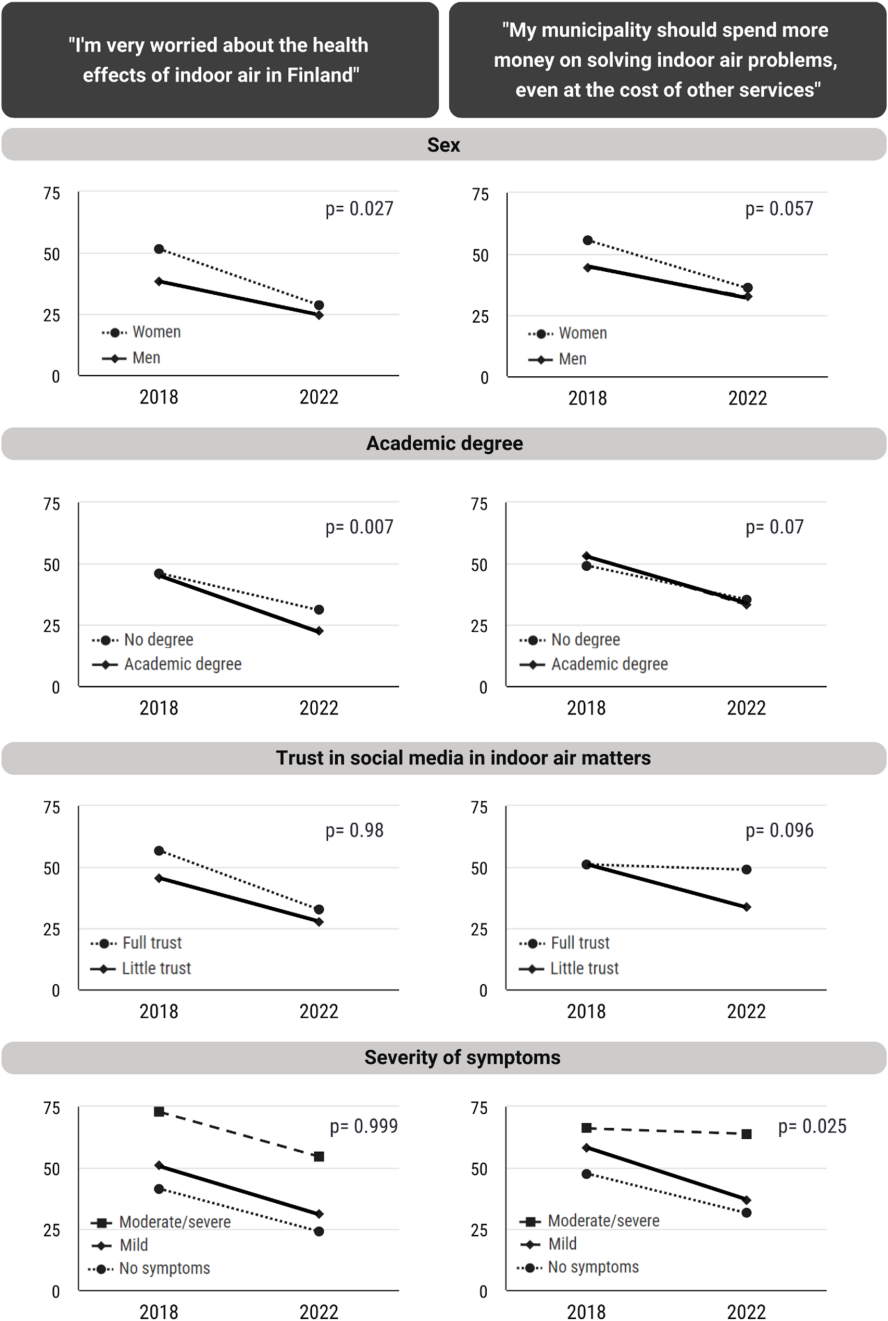
Differences in the proportional change of prevalence between subgroups from 2018 to 2022. *P*-values adjusted for age, sex, and education

Differences between surveys in terms of demographics, and the prevalence (in percentages) of symptoms and other characteristics were studied using binomial logistic regression – except for knowledge outcomes, for which multinomial logistic regression was used instead. In all tables, the outcome used in the analysis is the row variable, for example sex or symptoms at home. The *p*-values and odds ratios refer to the difference between 2018 and 2022. Proportional changes in prevalence were calculated as the difference of prevalence in 2022 and 2018, divided by the prevalence in 2018. Selected outcomes related to symptoms prevalence, perceived indoor air quality, risk perceptions, views on indoor air and knowledge with the largest changes from 2018 to 2022 were chosen for subgroup analysis (Fig. 1, and Additional file 3). Analyses were adjusted for age, sex, and education. In order to validate our findings in another dataset, the changes in the prevalence of indoor air-related symptoms and physician visits from NSI were compared with comparable data from FH 2017 and HFS 2023 (Table 3). Statistical analyses were conducted in IBM SPSS 28.0 for Windows (SPSS, Illinois, Chicago, IL).

## Results

A few statistically significant changes were seen in demographic characteristics between NSI 2018 and 2022 (Table 1). The data from 2022 had significantly fewer women and more people with an academic degree. The proportion of individuals living in an urban area also increased while the number of people in owner-occupied housing decreased. Trust in state or municipal officials in matters of indoor air increased, while trust in social media decreased.

**Table 1:** Change in demographic characteristics and trust between National surveys on indoor air 2018 and 2022

	2018 (*n* = 1754*), %	2022 (*n* = 1581*), %	*p*-value
Sex			
* Female*	56.8	53.1	0.007
Age			
* 25–34*	20.4	20.7	
* 35–49*	31.2	29.6	0.050
* 50–64*	48.5	49.7	
Marital status			
* Married*	51.5	51.3	> 0.1
Education			
* Academic degree*	39.1	47.3	< 0.001
Employment			
* Employed*	73.1	76.1	> 0.1
Place of residence			
* Urban area*	65.2	69.5	0.045
Owner-occupied housing	77.7	75.5	0.028
Form of housing			
* Single-family home*	48.6	46.5	> 0.1
Smoking	20.4	17.6	> 0.1
Trust in these authorities in matters of indoor air**			
* State or municipal officials*	27.5	37.3	< 0.001
* Media (TV*,* radio*,* etc.)*	18.5	21.1	> 0.1
* Social media*	5.0	3.1	0.011

*P*-values refer to the difference between 2018 and 2022. *P*-values are adjusted for age, sex, and education

*Due to missing data, the number of respondents varies between *n*=1754-1797 in 2018 and between *n*=1581-1605 in 2022

**trust to full trust

### Changes in indoor air-related symptoms

Based on NSI 2018 and 2022, indoor air-related symptoms at work decreased from 2018 to 2022 (Table 2). This trend was observed both among women, with a decrease from 26.2% to 21.0%, and men, with a decrease from 15.0% to 10.7% (gender differences shown only in the text). No such decline was seen in symptoms at home, neither among women (prevalence 7.2% in 2018 and 8.9% in 2022), nor among men (6.1% in 2018 and 7.4% in 2022). The frequency of visiting a physician due to indoor air symptoms also decreased. Again, this was seen both among women, from 4.6% to 2.8%, and among men, from 2.6% to 1.3%.

**Table 2:** Change in prevalence of symptoms and perceived indoor air quality between National surveys on indoor air 2018 and 2022

	2018 (*n* = 1745*), %	2022 (*n* = 1490*), %	Proportional change	OR (95%CI)	*p*-value
Symptoms at home**	6.7	8.2	22%	1.24 (0.95–1.62)	> 0.1
Symptoms at work**	21.3	16.2	−24%	0.73 (0.60–0.87)	< 0.001
Physician visit**	3.7	2.1	−43%	0.59 (0.38–0.91)	0.018
Sick leave**	2.5	1.7	−32%	0.65 (0.39–1.08)	0.094
Severity of symptoms**					
No symptoms	77.4	79.3	2%		
Mild symptoms	12.6	12.7	1%		
Moderate symptoms	8.2	6.4	−22%	0.77 (0.60–0.99)	0.044
Severe symptoms	1.9	1.6	−16%		
Self-perceived sensitivity to experience symptoms from poor indoor air quality***	12.0	11.4	−5%	0.97 (0.78–1.20)	> 0.1
Good self-reported indoor air quality at home****	84	85.4	2%	1.10 (0.91–1.33)	> 0.1
	*n* = 1324	*n* = 1292			
Good self-reported indoor air quality at work/place of study****	58.2	65.5	13%	1.30 (1.10–1.53)	0.002

*OR* odds ratio, *CI* 95% confidence interval

*P*-values and odds ratios refer to the difference between 2018 and 2022. Odds ratio, its 95% confidence interval, and the *p*-value are adjusted for age, sex, and education

*Due to missing data, the number of respondents varies between *n*=1745-1772 in 2018 and between *n*=1490-1594 in 2022

**either at home or at work due to indoor air in the last 12 months

***much or very much more easily than other people

****good or very good

Very similar changes in indoor air-related symptoms at work and visits to a physician were observed between FH 2017 and HFS 2023 (Table 3), as was seen in NSI 2018 and 2022 (Table 2), both among men and women.

**Table 3:** Changes in prevalence of indoor air-related symptoms and physician visits due to these symptoms between finhealth 2017 and healthy Finland 2023 surveys

	2017 (*n* = 3495*), %	2023 (*n* = 2832*), %	Proportional change	OR (95%CI)	*p*-value
Symptoms at home**	4.8	4.1	−15%	0.85 (0.66–1.09)	0.200
Symptoms at work**	17.0	12.3	−28%	0.68 (0.58–0.81)	< 0.001
Physician visit**	2.9	1.4	−52%	0.49 (0.32–0.76)	0.001

*OR *odds ratio, *CI* 95% confidence interval

*P*-values and odds ratios refer to the difference between 2018 and 2022. Odds ratio, its 95% confidence interval, and the *p*-value are adjusted for age, sex, and education

*Due to missing data, the number of respondents varies between *n*=3495-3512 in 2017 and between *n*=2832-2851 in 2023

**in the last 12 months

The prevalence of mild, moderate, or severe symptoms either at home or at work decreased from 22.6 to 20.7%, but the decrease was not statistically significant (Table 2). Regarding severity of symptoms at home or at work, while there were no fewer with mild symptoms, the combined prevalence of moderate to severe symptoms fell from 10.0 to 8.0%. The proportion of respondents who perceived the quality of indoor air at their home to be good stayed about the same, but self-perceived good indoor air quality at work increased from 58.2 to 65.5%. Self-perceived sensitivity to experience symptoms from poor indoor air quality did not change significantly.

The prevalence of telecommuting was measured with an added question to the 2022 survey on how much time they spend at the workplace each week. Of those spending at least 30 h per week at the workplace 21.9% reported getting symptoms at work. Of those spending less time at the workplace 17.9% reported symptoms at work. In 2018, 25.9% of employed people reported symptoms (Data not shown).

### Changes in risk perceptions, views, and satisfaction with municipalities

Compared to 2018, in 2022 fewer people thought that traffic pollution, moisture damage, other indoor impurities, and mobile phone radiation are large health risks for the Finnish population in general (Table 4). With other factors, however, such as small-scale biomass burning, traffic noise pollution, passive smoking, or radon in indoor air, there were no major changes in risk perceptions. In 2022, fewer participants were very worried about the health effects of indoor air. Agreement with other similar statements, such as whether indoor air problems are discussed a lot or whether more money should be spent on indoor air problems, also decreased. Out of the eight statements, six changed statistically significantly — all in the direction suggesting less health worry about the health effects of poor indoor air quality.

**Table 4:** Change in risk perceptions and views on indoor air between National surveys on indoor air 2018 and 2022

	2018 (*n*=1753*), %	2022 (*n*=1578*), %	Proportional change	OR (95%CI)	*p*-value
Large perceived health risk for the population**					
* Traffic pollution*	15.0	10.7	-29 %	0.74 (0.60-0.92)	0.005
* Small-scale biomass burning*	3.3	3.9	18 %	1.28 (0.89-1.86)	>0.1
* Traffic noise pollution*	11.3	10.7	-5 %	1.04 (0.83-1.30)	>0.1
* Passive smoking*	18.5	16.7	-10 %	0.93 (0.77-1.12)	>0.1
* Radon in indoor air*	8.4	6.8	-19 %	0.84 (0.64-1.09)	>0.1
* Moisture damage at home*	28.6	19.3	-33 %	0.62 (0.53-0.73)	<0.001
* Moisture damage at work*	36.4	23.2	-36 %	0.55 (0.47-0.64)	<0.001
* Moisture damage in public buildings*	43.3	29.3	-32 %	0.56 (0.48-0.64)	<0.001
* Other indoor air impurities (chemicals, etc.)*	21.4	13.8	-36 %	0.62 (0.52-0.75)	<0.001
* Mobile phone radiation*	20.4	14.2	-30 %	0.67 (0.56-0.81)	<0.001
Agreement with statements about indoor air problems***					
* The officials in my municipality don't take indoor air problems seriously enough.*	34.4	24.4	-29 %	0.63 (0.54-0.74)	<0.001
* My municipality should spend more money on solving indoor air problems, even at the cost of other services.*	50.8	34.4	-32 %	0.51 (0.45-0.59)	<0.001
* Health hazards caused by indoor air problems are downplayed in Finland.*	62.6	47.5	-24 %	0.55 (0.48-0.64)	<0.001
* I have enough information about health risks related to indoor air.*	51.7	52.7	2 %	1.01 (0.88-1.16)	>0.1
* I have enough information on how to influence the indoor air quality in my own home.*	68.2	72.7	7 %	1.20 (1.03-1.40)	0.019
* I have enough information on how to act, when I suspect indoor air problems or have symptoms due to indoor air.*	60.8	63.8	5 %	1.11 (0.96-1.28)	>0.1
* Indoor air problems are discussed a lot in my circle of acquaintances.*	43.5	27.9	-36 %	0.51 (0.44-0.59)	<0.001
* I'm very worried about the health effects of indoor air in Finland.*	45.8	26.8	-41 %	0.45 (0.39-0.52)	<0.001
Satisfied with the indoor air-related actions of one's own municipality****	28.4	28.9	2 %	1.00 (0.86-1.16)	>0.1
Satisfied with the actions of the municipality in the following indoor air-related issues****					
* Quality of construction and maintenance*	23.5	32.7	39 %	1.57 (1.34-1.83)	<0.001
* Solving the indoor air problems of schools*	25.5	31.5	24 %	1.33 (1.14-1.55)	<0.001
* Informing on indoor air issues*	20.5	22.3	9 %	1.09 (0.92-1.30)	>0.1
* Housing inspections by municipal health inspectors*	12.3	15.8	28 %	1.37 (1.12-1.67)	0.002

*OR* odds ratio, *CI* 95% confidence interval

*P*-values and odds ratios refer to the difference between 2018 and 2022. Odds ratio, its 95% confidence interval, and the *p*-value are adjusted for age, sex, and education

*Due to missing data, the number of respondents varies between *n*=1753-1773 in 2018 and between *n*=1578-1593 in 2022

**Large or very large risk

***Agree or fully agree

****satisfied or very satisfied

There was a significant increase in satisfaction with the indoor air-related actions of one’s own municipality in 3 out of the 4 specific actions asked. However, in a separate question asking about general satisfaction with the indoor air-related actions of one’s own municipality, the prevalence did not change (Table 4).

### Changes in knowledge

In 2018, respondents’ knowledge of the health effects of indoor air (Table 5) was at many points contrary to the messages promoted by the programme (bolded in Table 5). That was still the case in 2022, but there had nevertheless been some improvement: in all cases but one, agreement with the option, which had been promoted by the programme, had become more common.

**Table 5:** Changes in knowledge of the health effects of indoor air between National surveys on indoor air 2018 and 2022. Options promoted by the programme are bolded

		2018 (*n* = 1767*), %	2022 (*n* = 1584*), %	Proportional change	*p*-value (df = 2)
Statements about the health effects of indoor air					
*It is difficult to assess whether a person’s respiratory symptoms are caused by the indoor air of a building or something else.*	**Agree**	**58.9**	**63.4**	**8%**	
	Neither/can’t say	16.9	18.2	8%	< 0.001
	Disagree	24.2	18.4	−24%	
*Symptoms are the best gauge of indoor air quality.*	Agree	45.2	36.8	−19%	
	Neither/can’t say	28.8	32.0	11%	< 0.001
	**Disagree**	**25.9**	**31.2**	**20%**	
*The assessment of indoor air impurities should be based primarily on the examination of the building and other measurements rather than symptoms.*	**Agree**	**43.7**	**48.2**	**10%**	
	Neither/can’t say	24.2	27.2	12%	< 0.001
	Disagree	32.1	24.5	−24%	
*Even minor moisture damage is so harmful to health that immediate action has to be taken.*	Agree	63.3	54.6	−14%	
	Neither/can’t say	19.8	24.2	22%	< 0.001
	**Disagree**	**16.9**	**21.2**	**25%**	
*The appearance of actinomyces is a certain sign of harmfulness of indoor air.*	Agree	63.1	58.4	−7%	
	Neither/can’t say	33.4	36.7	10%	0.022
	**Disagree**	**3.6**	**4.9**	**36%**	
*The majority of indoor air symptoms are transient.*	**Agree**	**9.0**	**9.7**	**8%**	
	Neither/can’t say	27.8	33.5	21%	0.002
	Disagree	63.2	56.9	−10%	
*Mere concern about indoor air quality can effect symptoms similar to indoor air symptoms.*	**Agree**	**31.6**	**36.7**	**16%**	
	Neither/can’t say	34.2	36.7	7%	< 0.001
	Disagree	34.1	26.6	−22%	
*Spending time in a moisture-damaged building may lead to indoor air impurities preventing the use of most buildings.*	Agree	52.3	46.2	−12%	
	Neither/can’t say	33.8	39.7	17%	0.002
	**Disagree**	**13.9**	**14.2**	**2%**	
*It is possible to recover from sensitivity to get symptoms from indoor air.*	**Agree**	**15.7**	**17.5**	**11%**	
	Neither/can’t say	45.2	49.0	8%	0.029
	Disagree	39.1	33.5	−14%	
*Buildings can be clearly divided into healthy and problematic buildings.*	Agree	36.7	33.8	−8%	
	Neither/can’t say	35.1	39.2	12%	0.021
	**Disagree**	**28.3**	**27.1**	**−4%**	

*P*-values and refer to the difference between 2018 and 2022. *P*-values are adjusted for age, sex, and education

*Due to missing data, the number of respondents varies between *n*=1767-1778 in 2018 and between *n*=1584-1594 in 2022

The largest changes observed in Tables 2, 4 and 5, for example health worry and moisture damage-related risk perception, were then explored in different subgroups (Fig. 1, and Additional file 3). Women tended to have larger proportional changes than men, as did those with an academic degree compared to those without an academic degree. However, only a few of the studied differences between the subgroups had a *p*-value below 0.1. There was a particularly large difference in change between men and women in health worry about the effects of indoor air. In 2018, 51.6% of women were worried compared to 38.2% of men (Fig. 1), while in 2022, the difference had decreased significantly (women 28.6% vs. men 24.7%). Similar changes were seen with the level of education: in 2018, those with an academic degree had more often a lot of discussion in their circle of acquaintances about indoor air problems (46.9% with a degree vs. 41.2% without), while in 2022, the order had changed (26.1% vs. 29.8%). Those with high trust in social media tended to have smaller positive changes or even negative changes compared to those who had less trust in social media. Similarly, those with moderate to severe severity of symptoms were often resistant to change, though that was not universal: e.g., they had a higher increase in good indoor air at work than average. The analyses on severity of indoor air-related symptoms were also repeated using severity of functional impairment due to indoor air-related symptoms [3]. This produced very similar, although slightly weaker associations (data not shown).

We performed similar subgroup analyses as in Fig. 1 also by age of the respondents, i.e. those above and below the age of 45 years, but observed no statistically significant differences between those two age groups (data not shown).

## Discussion

This study examined the changes in the prevalence of indoor air-related symptoms, perceived indoor air quality, health worry, and other views on indoor air between the National Survey on Indoor Air (NSI) conducted in 2018 and 2022 with repeated random samples of working-age Finns. In accordance with the objectives of the Finnish Indoor Air and Health Programme 2018–2028, symptoms at work decreased and satisfaction with indoor air quality at work increased, though similar changes were not seen at home. This finding on symptoms was also found in two other large national population-based surveys in Finland, FinHealth 2017 (FH) and the Healthy Finland Survey (HFS) 2023. A major decrease was observed in health worry regarding indoor air effects. Knowledge of indoor air health effects also somewhat improved. Changes tended to be stronger among women, those with an academic degree, those with less trust in social media, and those with less severe symptoms.

The interpretation of the present results is confounded by several factors outside of the effects of the programme. The most obvious factors are recent geopolitical disturbances, such as the COVID-19 pandemic. The pandemic prompted many to work from home, a trend that has not completely reversed. Working from home is likely to affect especially reported symptoms at work and satisfaction with indoor air quality at work. In the present study, telecommuting was measured with an added question to the 2022 survey about the amount of time spent working on-site each week. While working less time on-site decreased symptoms at work, the results suggest that symptoms also decreased for those who did not telecommute in 2022.

In addition, municipalities have also greatly invested in improving the indoor air quality of their buildings during 2019–2023 [19]. This has been implemented through the renovation and redevelopment of, especially, schools and daycare centres. In addition, operating models for indoor air issues have been created and updated. Following the implementation of these measures, the municipalities report a decrease in severe indoor air issues from 2019 to 2023. Another factor is the Russian invasion of Ukraine, which may have overshadowed indoor air issues, e.g. in public media and political discussions.

Media has been suggested to have an impact on symptom reporting [20]. News stories of adverse environmental health effects have been shown to increase the number of symptom reports related to idiopathic environmental intolerances [21–23]. A recent analysis of media coverage examined how indoor air was portrayed in Finnish editorial media from 2017 to 2022 [12]. The frequency of indoor air-related news steadily declined over this period from a yearly average of 5859 instances in 2017–2018 to 2542 instances in 2021–2022. Also, the proportion of negative news (e.g. news that overemphasises health risks) halved from 30 to 15% during the same period. Hence, it is quite possible that the reduction in indoor air news overall, and negative news specifically, has alleviated health worry and symptom reports. The observed changes in the frequency and tone of indoor air-related news are, in turn, potentially influenced by the actions of the programme.

Although the independent effect of the programme on the observed changes is impossible to estimate, we consider it likely that the programme has contributed to the favourable changes seen.

The overall objective of the Finnish Indoor Air and Health programme is to reduce hazards to health and well-being linked to indoor environments in Finland. The main indicators used to monitor the attainment of this objective are changes in the prevalence of indoor air-related symptoms and satisfaction with indoor environments. Though the already relatively rare symptoms at home did not decrease, those experienced at work decreased by approximately one-fourth. We also observed a substantial decrease in health worry regarding the health effects of indoor air. Since perceived environmental risks have been shown to be associated with increased symptom reporting [24, 25], the decrease in health worry might have contributed to the symptom reduction. We also observed that the prevalence of perceived good indoor air quality at work increased by 13%, while remaining consistently at the same higher level at home. This difference might be explained by the ceiling effect, where the already high prevalence of good indoor air quality at home makes detecting changes more challenging compared to settings with a lower prevalence.

The first area of the programme (Area 1) aims to provide evidence-based information to the general public. The goal is that knowledge about the effects of indoor environments on health and well-being is improved. In 2018, the general public’s knowledge of the health effects of indoor was at many points contrary to current scientific knowledge [13]. Several consistent and significant changes, although small in size, were observed, which suggest that improvements have already occurred during the first phase of the programme.

As shown in the present paper, moisture damage is more commonly perceived as a large health risk to the Finnish population (Table 4) than particulate air pollution, radon, or passive smoking, which is in conflict with the burden of disease due to these factors [8]. We observed a roughly one-third decrease in the prevalence of those considering moisture damage a significant health risk, which surpassed decreases seen with other risk factors. Similarly, knowledge about indoor air-related issues improved. The decrease in indoor air-related worry and the improvement of knowledge may be partly the result of successful communication by experts linked to the programme and a decrease in negative indoor air-related news in the media [12]. The awareness of indoor air exposures and associated risk perceptions have proved to substantially influence perceived indoor air quality and symptom reporting [26, 27]. While we are not aware of any similar programme in the field of indoor air with surveys comparable to the present study, similar results have been achieved by other risk communication programs related to environmental risks, such as salmonella, asbestos, and flash floods [28–31], even though they have used more direct communication channels, such as television, radio, flyers, and billboards.

The focus of the second area of the programme (Area 2) is the management of problem situations in buildings linked to indoor environments. The goal for this area is to enhance residents’ trust in their own municipality’s capacity to address indoor air problems. Several actions have been undertaken toward this goal. In addition, municipalities have recently made substantial investments in improving indoor air quality in their buildings, and the number of severe indoor air problems has declined [32]. We observed that satisfaction with specific indoor air-related actions of the municipality increased by up to 39%. Even though satisfaction with the municipality increased based on specific questions, overall satisfaction with its indoor air-related actions remained unchanged. This may mean that the specific questions did not include all major factors affecting overall satisfaction. Alternatively, respondents may have replied to the question on overall satisfaction without sufficiently thinking of the specific areas asked later, which the municipalities have especially focused on and where improvements have taken place [32]. The response might also have been different had overall satisfaction been asked after the specific areas.

The observed changes were more pronounced among women, those with an academic degree, and those with less severe symptoms. Conversely, those with high trust in social media had smaller positive changes or even negative changes. A possible explanation for these dissimilar changes is a difference in health literacy, defined as the ability to comprehend health information [33]. It is expected that those with better health literacy are more affected by the programme, which has produced and published indoor air-related health information. Education is known to positively impact health literacy [34, 35], and some studies have also found men to have lower health literacy [36], but others have found no difference [35]. We observed that while changes occurred in the same direction for both women and men, women tended to have larger changes than men. This may be explained by a disparity in health literacy or by women tending to be more receptive to the health-related information disseminated through media [37], in which, as previously described, there has been a decrease in negative news stories about indoor air [12].

The Finnish Indoor Air and Health Programme was developed utilizing the experience gained from the past successful national programmes for controlling chronic respiratory diseases [38], such as the Asthma Programme 1994–2004 [14] and the Finnish Allergy Programme 2008–2018 [15]. Based on the past programmes, the goal was to take a holistic, science-based approach to solving indoor air problems, to reach a broad consensus with essential stakeholders, and to involve them in the implementation (Additional file 1). The programmes also underlined the importance of planning and focusing our actions carefully. They emphasised the need to target al.l interested parties, including the general public, for a nationwide impact. Although the programme was favourably received by, for example, most of the municipalities, medical doctors, newspapers, and businesses, there was some degree of pushback. In particular, the programme’s message that psychosocial aspects should also be considered in the management of indoor air problems encountered strong opposition especially on social media from segments of the public, some patients, and a few medical doctors. This indicates the need for continued efforts to educate professionals and to inform the public.

The Finnish real-world, long-term interventions on chronic respiratory diseases [38] have shown that national programmes can change attitudes, improve prevention and management, and reduce costs. After the aforementioned programmes, the disease burden of asthma and allergies decreased significantly. The promising findings of the present paper give us hope that similar outcomes may be achieved at the end of the Finnish Indoor Air and Health Programme 2018–2028. The positive results are also an indication to continue the programme as planned.

Strengths of this study include the large population-based random samples, which allowed us to examine a wide variety of indoor environment-related health factors. Despite the participation rates in NSI being low at 36% and 32%, they remained consistent, and only little participation bias was detected for indoor air-related symptoms when compared with the two other Finnish surveys with higher participation rates, FH 2017 and HFS 2023. The most significant limitation of our study is the COVID-19 pandemic, as discussed above. Many other societal changes, for example in terms of social media and its consumption, also complicate the interpretation of the data. The programme had no initiatives specifically related to the pandemic. We do not have information on how much the survey participants have been exposed to the programme, so we cannot analyse the effects of the programme on the individual level. Instead, we compare the effects of a population-based intervention over time using two independent sets of repeated cross-sectional studies design, which makes it practically impossible to accurately disentangle the causal factors behind the observed changes. In addition, the present results are only generalisable to the working age population and all of the data is based on self-report.

## Conclusions

There have been positive changes in several indoor air-related factors in the Finnish adult population between 2018 and 2022 during the employment of the Finnish Indoor Air and Health Programme 2018–2028. Symptoms related to indoor air at work and severe symptoms in general decreased, and the prevalence of perceived good indoor air quality at work increased. Health worry about indoor air-related issues decreased significantly, and knowledge of the health effects of indoor air somewhat improved. Although COVID-19 and other factors complicate the evaluation of the independent effect of the programme, we nevertheless find it likely that the Finnish Indoor Air and Health Programme 2018–2028 has contributed at least partly to the positive changes described in this paper.

## Supplementary Information

Additional file 1.

Additional file 2.

Additional file 3.

## Data Availability

Data will be shared upon reasonable request, when consistent with the ethical review statement.
