# Supplementary material for: Major changes in indoor air-related symptoms, health worry, and views between 2018 and 2022 in Finland
Source: BMC Public Health. 2025 Oct 10;25:3459. doi: 10.1186/s12889-025-24224-8 (PMC12512946; doi:10.1186/s12889-025-24224-8)
Supplement: Supplementary file 1 — Additional file 1. [file 12889_2025_24224_MOESM1_ESM.pdf]

Objectives and outcomes of the Finnish Indoor Air and Health Programme 2018–2022

In spring 2017, the Finnish Institute for Health and Welfare initiated the preparation of the Finnish Indoor Air and Health Programme (Lampi et al. 2020) together with collaborators, such as the Finnish Institute of Occupational Health, and stakeholders. The program drew on the experiences from past successful programmes in Finland, such as the Finnish Asthma Programme and the Finnish Allergy Programme (Erhola et al. 2019). The program was designed using the logical framework approach method in a total of eight workshops and other extensive planning meetings, together with stakeholder hearings and two new reviews.

The primary objective of the Indoor Air and Health Programme is to reduce indoor environment-related hazards to health and well-being in Finland. Already during the planning phase of the programme, it was recognised that simply reducing indoor air pollution is not enough, but that other measures would also be needed (Lampi et al. 2020). Therefore, in contrast to past prevention efforts, humans, not air pollution levels, are the central focus of the programme. The objectives of the program are to be achieved through measures in four areas: informing the general public (1), management of indoor air-linked problems in buildings (2), developing the treatment and support of people with indoor air-related symptoms and illnesses such as to improve their functional and work capacity (3), and education and training of professionals and other actors in indoor environment-related issues (4).

The programme was coordinated by the Finnish Institute of Health and Welfare, but all major decisions were done in the very active secretariat of the programme, which met monthly. The secretariat included representatives from major organizations working on indoor air and health in Finland: the two major governmental research institutes (Finnish Institute for Health and Welfare and Finnish Institute of Occupational Health), the two major patient organizations (The Organisation for Respiratory Health in Finland and The Finnish Allergy, Skin and Asthma Federation), two expert NGO (Finnish Lung Health Association and Finnish Society of Indoor Air Quality and Climate), and Ministry of Social Affairs and Health, which also provided funding for the programme (Lampi et al. 2020).

The fulfilment of the programme's objectives is monitored using predefined indicators. For the main objective and areas 1 and 2, progress is monitored through surveys, while for areas 3 and 4 progress is monitored through outcome indicators, i.e. the implementation of planned measures (Lampi et al. 2020). During the first five years of the Finnish Indoor Air and Health Programme, those measures were implemented that were evaluated during the planning phase as most important or most effective in relation to the objectives. The following describes the actions taken in the program, divided into the programme's four sub-areas, although many of the actions taken support several areas of the program.

Area 1: Influencing through information

To target risk communication actions and to evaluate the programme, a national population-based survey, the National Survey on Indoor Air 2018, was conducted at the beginning of the programme to determine Finnish people's perceptions of indoor air health risks (Lampi et al. 2019). Information on the health effects of indoor air pollutants and moisture damage was published and shared with the general public much more actively than before, based on the outputs of the different areas of the programme, and past and new research. The communications highlighted, among other things, that factors other than indoor air exposure affect indoor air symptoms, which makes symptoms an unreliable way to assess indoor air health and stated that many claims made in public about the

health effects of indoor air exposure, particularly moisture damage and mould, and "indoor air sensitivity" do not correspond to the current knowledge (Pekkanen et al. 2023). The tools used were carefully designed press releases for the general public, new online material banks such as frequently asked questions and updated websites, blogs, interviews and appearances in media, lectures and training, and discussions with stakeholders. Many of the topics of the programme received significant attention in the media (Meltwater 2023). For example, the main Finnish newspaper published two editorials praising the programme and promoting its goals (Editorial 2019, 2020)

Area 2: Problem situations in buildings

The management of problem situations in buildings has been improved by developing management processes, clarifying the responsibilities and roles of different actors, and developing the assessment of exposure conditions and health significance in indoor air situations. To guide the programme's work, several online surveys and workshops have been conducted with, among others, municipal property owners, employers and occupational safety representatives, indoor air groups, school health care, and maternity clinics. The focus of the work has been on introducing good practices to support working life and solve problems in buildings. These have included perspectives on prevention, on orientation to the use of buildings, and on the multifactorial nature of symptoms. Actions related to these have been directed, for example, at property owners for proactive property maintenance and management of situations related to the indoor environment, and school principals and daycare centre directors (Jalkanen et al. 2022a, Salmela et al. 2022, Salmela et al. 2023a). School health care has been given guidance to support groups of students and individual students in indoor air situations (Salmela et al. 2023b).

The indoor air guidelines about indoor environment assessment (Isokääntä et al. 2023) and health impact assessment (Reijula et al. 2023) were updated to reflect the current state of knowledge. This work was based on extensive reviews of various indoor air pollutants and their health effects (Tuomi et al. 2020, Wallenius et al. 2021, Juntunen et al. 2022a, Leppänen et al. 2022, Viljamaa et al. 2022, Wallenius et al. 2023a, Yli-Tuomi et al. 2023). Municipal actions have been supported by, among other things, updated guidelines on the use of measures to ensure safe use of premises (Juntunen et al. 2022b) and the Indoor Air Association's good indoor air recommendation on the use of ventilation outside building operating hours (Municipal Indoor Air Network 2019). Workplaces have been supported with guidelines on post-repair cleaning and cleaning of movable property (Isokääntä et al. 2022).

To support actors at workplaces, an operational model has been developed for supporting work capacity in indoor air situations (Keränen et al. 2021, Finnish Institute of Occupational Health 2024a). In addition, reviews have been written on environmental factors that support well-being and work performance (Wallenius et al. 2023b) and on the use of alternative and specially cleaned premises in cases of indoor air problems (Lappalainen et al. 2020). The new guidelines are not only intended for property owners, school operators, and workplaces; the program has also created guidelines for residents on factors affecting housing health (Salmela 2023c), cleaning apartments after moisture damage and mould (Juntunen et al. 2021) and an operating model for managing indoor air situations (Jalkanen et al. 2022b).

Area 3: Care and support for people

Area 3 compiles information for all those who experience symptoms in indoor environments and are adversely affected by indoor environments and strengthens counselling and peer activities. When the program was launched, support was provided for the launch of the HUS rehabilitation clinic for patients with symptoms in indoor environments whose work and functional capacity has been seriously impaired, and for developing a web-based exercise programme to support the treatment of prolonged symptoms. In addition, a leaflet (Finnish Institute of Occupational Health 2020a) and online materials on factors affecting symptoms and treatment methods were produced for both healthcare professionals and patients, and guidelines were produced on restoring functional capacity in cases of prolonged symptoms (Finnish Institute of Occupational Health 2024b).

The programme structure has included healthcare expert group from different fields of medicine and different parts of Finland, whose task has been to support the implementation of the programme. In addition to regular meetings, the group has prepared two statements. One of the statements concerned the use of the term "indoor air disease" and the other concerned special clean rooms and their implementation (Finnish Indoor Air and Health Program Healthcare Expert Group 2020 and 2021).

In the area of treatment and support, studies have been conducted on the current state of treatment and service pathways in healthcare (Renkola et al. 2020, Vuokko et al. 2021, Granlund et al. 2024) and on indoor air symptoms that severely limit functional capacity (Frilander et al. 2018). In addition, recommendations have been piloted and drawn up for the introduction of an operating model for supporting work capacity (Finnish Institute of Occupational Health 2024a), and articles have been written on various topics related to health and symptoms, which have been published in, among others, the Duodecim journal and the Finnish medical journal (e.g., Pekkanen et al. 2020, Louhiala et al. 2020, Pekkanen et al. 2023).

Area 4: Education

Extensive training has been provided in healthcare, and information packages and tools have been compiled to support treatment and rehabilitation. At the beginning of the programme, training courses were launched for physicians and other healthcare professionals in each hospital district.

Training materials have also been produced for the TerveyskyläPro web based platform and video library for use by all healthcare professionals, and a training package has been compiled for universities of applied sciences in the healthcare field. In addition, the training needs of both physicians and nurses in the care of patients with indoor air symptoms have been identified (Renkola et al. 2020, Vuokko et al. 2021).

Training has also been increased in workplaces, and for indoor climate experts, occupational health professionals, employees, and those who support them. Training for the above-mentioned parties has been provided through webinars, seminars, and guidelines and materials compiled on websites, such as open learning materials: Indoor Air at Work - online material (Finnish Institute of Occupational Health 2020b).

References

Editorial (2019). ‘Kosteusongelmat lietsovat pelkoja’ (in Finnish). Helsingin Sanomat 6.11.2019

Editorial (2020). ‘Sisäilmaongelma on liian yleinen selitys’ (in Finnish). Helsingin Sanomat 16.11.2020

Erhola, M., T. Vasankari, V. Jormanainen, S. Toppila-Salmi, J. Herrala, and T. Haahtela. (2019). 25 years of respiratory health in Finland. Lancet Respir Med 7:e16

Frilander H, Karvala K, Sainio M, Vuokko A. (2018) Toimintakykyä rajoittava sisäilmaoireisto. Työterveyslaitos. https://urn.fi/URN:ISBN:9789522618726

Granlund H, Renkola H, Vasankari T, Salmela A. Terveydenhuollon näkemyksiä sisäilmasta oireilevan potilaan hoidosta. Terveyden ja hyvinvoinnin laitos, Tutkimuksesta tiiviisti 30/2024. https://urn.fi/URN:ISBN:978-952-408-337-9

Isokääntä P Kanerva T, Korenius P, Liukkonen T, Manninen T, Rautiala S (2022) Ohje korjausten jälkeiseen siivoukseen ja irtaimiston puhdistukseen työpaikoilla. Työterveyslaitos 2022 https://urn.fi/URN:ISBN:978-952-391-056-0

Isokääntä P ja Rautiala S (2023) Sisäilmastoselvitys ja olosuhdearviointi: Ohje työpaikkojen sisäilmastoselvityksiä ja olosuhdearviointeja tekeville. Työterveyslaitos, Tietoa työstä. https://urn.fi/URN:ISBN:978-952-391-037-9

Jalkanen K, Salmela A, Hyvärinen A (2022a) Sisäilmatilanteen selvitysprosessi koulussa ja päiväkodissa. THL, Tutkimuksesta tiiviisti: 2021_032. https://urn.fi/URN:ISBN:978-952-343-672-5

Jalkanen K, Salmela A, Hyvärinen A (2022b) Toimintamalli sisäilmatilanteiden hoitamiseen asunnoissa. THL, Tutkimuksesta tiiviisti: 2022_044. https://urn.fi/URN:ISBN:978-952-343-936-8

Juntunen M, Jalkanen K, Salmela A, Leppänen H, Hyvärinen A (2021) Ohje asuntojen kosteus- ja mikrobivauriokorjausten jälkeiseen siivoukseen ja irtaimiston puhdistamiseen. THL, Tutkimuksesta tiiviisti: 2021_069. https://urn.fi/URN:ISBN:978-952-343-767-8

Juntunen M, Salmela A, Jalkanen K, Hovi H, Wallenius K, Hyvärinen A (2022a) Haihtuvat orgaaniset yhdisteet asunnoissa: Pitoisuustasot, yleisimmät yhdisteet ja terveysvaikutukset. THL, Työpaperi: 2022_005. https://urn.fi/URN:ISBN:978-952-343-809-5

Juntunen M, Salmela A, Jalkanen K, Leppänen H, Hyvärinen A (2022b) Käyttöä turvaavat toimenpiteet. THL, Tutkimuksesta tiiviisti: 2022_031. https://urn.fi/URN:ISBN:978-952-343-894-1

Kansallisen sisäilma ja terveys -ohjelman terveydenhuollon asiantuntijaryhmä (2020). ”Sisäilmasairaus” termin käytölle ei ole lääketieteellisiä perusteita: oireilevia tulee auttaa tukeutuen parhaaseen lääketieteelliseen tietoon. 20.8.2020 https://urn.fi/URN:NBN:fi-fe202103288617

Kansallisen sisäilma ja terveys -ohjelman terveydenhuollon asiantuntijaryhmä (2021). Sisäympäristöissä oireilevien siirto erityispuhtaisiin tiloihin: riskit ja selvitystarpeet. 30.03.2021 https://urn.fi/URN:NBN:fi-fe202103238140

Keränen H, Juvonen-Posti P, Wallenius K, Kinnari T, Vuokko A, Sainio M (2021). Työkyvyn tuen käytännöt työpaikoille sisäilmatilanteisiin. Moninäkökulmaisen toimintamallin yhteiskehittäminen asiantuntijoiden kysely- ja työpaja-aineistojen avulla. Työterveyslaitos, Tietoa työstä. https://urn.fi/URN:ISBN:978-952-261-987-7

Kuntien sisäilmaverkosto (2019). Julkisten palvelurakennusten ilmanvaihdon käytön yleisohje ja yleisohjeen perustelumuistio. Sisäilmayhdistyksen Hyvä sisäilma -suositus 1, 03/2019

Lampi J, Salmela A, Ung-Lanki S, Tuoresmäki P, Pekkanen J. (2019) Kansallinen sisäilmakartoitus 2018: aineisto ja menetelmät. Tutkimuksesta tiiviisti 39, 2019. Terveyden ja hyvinvoinnin laitos, Helsinki. https://urn.fi/URN:ISBN:978-952-343-397-7

Lampi J, Hyvärinen A, Erhola M, Haahtela T, Haukipuro K, Haverinen-Shaughnessy U, Jalkanen K, Karvala K, Lappalainen S, Reijula K, Rämö H, Sainio M, Salmela A, Salminen M, Vasankari T, Pekkanen J. Healthy people in healthy premises: the Finnish Indoor Air and Health Programme 2018-2028. Clin Transl Allergy. 2020 Jan 17;10:4. doi: 10.1186/s13601-020-0308-1

Lappalainen S, Rautiala S, Sainio M, Kinnari T, Hirvonen S. (2020) Väistö- ja erityispuhtaiden tilojen käyttö työpaikkojen sisäilmasto-ongelmatilanteissa: sisäilmasto- ja tilaratkaisuihin liittyvien toimintatapojen nykytilanne. Työterveyslaitos. https://urn.fi/URN:ISBN:9789522619761

Leppänen H, Jalkanen K, Salmela A, Hyvärinen A. (2022) Mikrobikasvuston selvittämiseen käytettävät menetelmät kosteusvauriokohteissa: kirjallisuuskatsaus. THL, Työpaperi: 2022_056.

Louhiala P, Pekkanen J, Elovainio M, Sainio M. (2020) Nosebon monet kasvot. Duodecim 2020;136:1333–8.

Meltwater (2023) Kansallinen sisäilma ja terveys -ohjelma: Medianäkyvyyden analyysi vuosina 2017–2022. https://thl.fi/tutkimus-ja-kehittaminen/tutkimukset-ja-hankkeet/kansallinen-sisailma-ja-terveys-ohjelma-2018-2028/ohjelman-tuotokset

Pekkanen J, Hyvärinen A, Sainio M, Erhola M, Haahtela T, Haverinen-Shaughnessy U, Haukipuro K, Jalkanen K, Karvala K, Lappalainen S, Reijula K, Rämö H, Salmela A, Salminen M, Vasankari T, Lampi J. (2020) Kansallinen sisäilma ja terveys -ohjelma 2018–2028: Parempaa terveysvaikutusten arviointia ja potilaiden hoitoa. Suomen Laakaril 2020;75:1290-95

Pekkanen, Juha, Anniina Salmela, Anne Hyvärinen, Anne M Karvonen, Hanna Leppänen, Tuula Vasankari, Aki Vuokko, Kaisa Wallenius, Kati Huttunen. (2023) Faktantarkistusta: sisäilma ja terveys, Duodecim, 139: 31-37

Reijula K, Korenius P, Keränen H, Tulenheimo-Eklund E, Vuokko A, Sainio M (2022) Terveydellisen merkityksen arviointi sisäilmatilanteissa. Työterveyslaitos, Tietoa työstä. https://urn.fi/URN:ISBN:978-952-391-047-8

Renkola H, Aro M, Heinikari T, Varankari T (2020) Selvitys sisäympäristössä oireilevien hoito- ja palvelupolkujen nykytilasta perusterveydenhuollossa ja erikoissairaanhoidon keuhkoklinikoissa. Filha ry

Salmela A, Jalkanen K, Hyvärinen A (2022) Ohjeistus rehtoreille sisäilmatilanteeseen. THL, Tutkimuksesta tiiviisti: 2022_062. https://urn.fi/URN:ISBN:978-952-408-004-0

Salmela A, Jalkanen K, Hyvärinen A (2023a) Ohje päiväkodin johtajille sisäilmatilanteeseen. THL, Tutkimuksesta tiiviisti: 2023_009. https://urn.fi/URN:ISBN:978-952-408-038-5

Salmela A, Jalkanen K, Hyvärinen A (2023b) Ohjeita koulutyöhön sisäilmatilanteessa. THL, Tutkimuksesta tiiviisti: 2023_033. https://urn.fi/URN:ISBN:978-952-408-116-0

Salmela A (2023c) Huomioi energiasäästötoimien vaikutus asumisterveyteen. THL, Tiedä&Toimi.

Tuomi T, Wallenius K, Mahiout S, Rautiala S, Lappalainen S (2020) Teolliset mineraalikuidut toimistotyyppisissä työtiloissa: Esiintyminen, altistumisen arviointi, terveysvaikutukset ja päästöjen hallinta. Työterveyslaitos, Tietoa työstä. https://urn.fi/URN:ISBN:9789522619167

Työterveyslaitos (2020a). Tietokortti: Tietoa oireilusta sisäympäristössä terveydenhuollon ammattilaisille. Kansallinen sisäilma ja terveys -ohjelma. https://www.julkari.fi/handle/10024/149143

Työterveyslaitos (2020b). Sisäilma työpaikalla -oppimateriaali. Saatavilla www.ttl.fi/sisailmatyopaikalla

Työterveyslaitos (2024a) Työkyvyn tuki sisäilmatilanteessa. Saatavilla: https://www.ttl.fi/teemat/tyohyvinvointi-ja-tyokyky/sisailma/tyokyvyn-tuki-sisailmatilanteissa.

Työterveyslaitos (2024b) Sisäilma, terveys ja oireilu -verkkosivut Saatavilla: https://www.ttl.fi/teemat/tyohyvinvointi-ja-tyokyky/sisailma/sisailma-terveys-ja-oireilu

Viljamaa A, Leppänen H, Jalkanen K, Salmela A, Hyvärinen A (2022) Kosteusvaurioindikaattorimikrobien rooli mikrobikasvuston määrittämisessä rakennusmateriaalista: aineistokatsaus. THL, Työpaperi: 2022_053. https://urn.fi/URN:ISBN:978-952-343-987-0

Vuokko A, Keränen H, Sainio M, Jokela P, Tulenheimo-Eklund E, Juvonen-Posti P, Reijula K (2021) Työntekijöiden oireilu sisäympäristössä: työterveyshuollon näkökulma. Työterveyslaitos, Tietoa työstä. https://urn.fi/URN:ISBN:9789522619563

Wallenius K, Hovi H, Mahiout S, Remes J, Rautiala S, Jokela P, Leino K, Liukkonen T (2021) Haihtuvat orgaaniset yhdisteet toimistotyyppisissä työympäristöissä: Päästölähteet, mittausmenetelmät, pitoisuustasot ja terveysvaikutukset. Työterveyslaitos, Tietoa työstä. https://urn.fi/URN:ISBN:9789522619570

Wallenius K, Korkalainen M, Porras S, Hovi H, Holma S, Ahtinen S, Koponen J, Huttunen K, Rantakokko P (2023a) Sisäympäristöissä esiintyvät puolihaihtuvat orgaaniset yhdisteet (SVOC). Väestön altistuminen ja terveysriskit. Työterveyslaitos, Tietoa työstä. https://urn.fi/URN:ISBN:978-952-391-114-7

Wallenius K, Malve-Ahlroth S, Keränen H, Haapakangas A (2023b) Hyvinvointia ja työn sujumista tukevat sisäympäristötekijät toimistossa. Työterveyslaitos, Tietoa työstä. https://urn.fi/URN:ISBN:978-952-391-082-9

Yli-Tuomi T ja Siponen T (2023) Katsaus sisätilojen PM2.5- ja PM10-hiukkasista. THL, Työpaperi 2023_022. https://urn.fi/URN:ISBN:978-952-408-117-7
