# Supplementary material for: Major changes in indoor air-related symptoms, health worry, and views between 2018 and 2022 in Finland
Source: BMC Public Health. 2025 Oct 10;25:3459. doi: 10.1186/s12889-025-24224-8 (PMC12512946; doi:10.1186/s12889-025-24224-8)
Supplement: Supplementary file 2 — Additional file 2. [file 12889_2025_24224_MOESM2_ESM.pdf]

TERVEYDEN JA
HYVINVOINNIN LAITOS

National Institute for Health and Welfare (THL), Environmental Health, P.O. Box 95, 70701 Kuopio, Finland

NATIONAL INDOOR AIR SURVEY 2018

FILLING INSTRUCTIONS

First, read the whole question carefully. Some of the questions provide more detailed answers.

To answer, tick the box x for the correct or most appropriate option, or type the requested information in the space allocated to it. Preferably use a ballpoint pen or pencil, but not a pencil.

If you accidentally mark the wrong box, black the wrong box completely and
tick the box in the right box.

For each question, choose only one of the most appropriate options.

BACKGROUNDINFORMATION

1. **Age**

2. **Sex**

3. **What's your marital status?**

    Married or in a registered partnership
    Cohabiting
    Unmarried
    Separated or divorced
    Widow

4. **What's your training?**
    Mark the highest level of training you have completed.

    Primary school or comprehensive school
    Middle school
    Vocational school or equivalent
    Gymnasium
    Postsecondary degree
    Bachelor's degree (polytechnic or equivalent)
    Master's degree (Master's or equivalent)

5. **Are you currently mainly** (choose only one option)

    Receiving salary or entrepreneur/self-employed (including unpaid work in the family business, apprenticeships and paid trainees);
    Unemployed
    Student
    Retired
    Housewife or father-at-home
    Something else

6. **What's your profession?** (If you are currently retired or unemployed, mark the occupation in which you last worked.)

____

HEALTH

7. **How is your health currently**

Good
Quite good
Intermediate
Quite bad.
Bad

8. **Have you had any of the following health problems diagnosed by your doctor** in the last 12 months**?**

Response options: Not 1 , Yes 2

Heart failure
Coronary heart disease
Sleep apnea
Diabetes
Asthma
Allergic rhinitis (e.g. hay fever)
Atopic dermatitis
Rheumatoid arthritis
Other joint disease
Depression
Other psychiatric illness

Chronic fatigue syndrome
Fibromyalgia
Irritable bowel syndrome
Chronic pain syndrome
Fragrance sensitivity
Multiple chemical sensitivity
Sensitivity to indoor air pollutants
Noise sensitivity
Electrical sensitivity

9. **Let's say your ability to work has scored 10 points at best. What score would you give for your current ability to work?** (tick the number corresponding to your ability to work)

completely incapacitated ……………work capacity at its best
0 1 2 3 4 5 6 7 8 9 10

INDOOR AIR-RELATED SYMPTOMS

10. Symptoms or illness related to indoor air

Response options: No yes, in the last 12 months yes, last time more than a year ago

Have you ever had any symptoms from indoor air at home?

Have you ever had any symptoms from indoor air at your workplace?

Have you ever been examined or treated by a doctor for symptoms or illness, which is suspected to be mainly caused by poor indoor air?

Have you even been on sick leave due to symptoms or illness, which is suspected to be mainly caused by bad indoor air?

11. How severe symptoms have you experienced from indoor air at home or work/place of study in the last 12 months?

If you have not experienced any symptoms of indoor air, please proceed to question 16

Response options:
No symptoms at all
Mild symptoms
Moderate symptoms
Severe symptoms
Very severe symptoms

Home
Work/study place
Other building, what? ____

12. What symptoms have you experienced in connection with indoor air and how often in the last 12 months?

Response options:
Not at all.
Once or twice a year
Once or twice a month
Once or twice a week
Almost every day

Eye disorders
Nasal congestion
Hoarseness
Dyspnoea
Cough
Skin symptoms

Joint symptoms
Tingling of the limbs
Headache
Dizziness
Nausea
Fatigue
Brain fog
Palpitation

Sweating
Urinary retention difficulties
Diarrhea
Fever

13. Have these symptoms made it difficult to work, manage your home affairs or get along with other people in the last 12 months?

Not at all
Quite a bit
Moderately
Quite a lot.
Very much

14. In the last 12 months, have you taken the following measures to avoid indoor air symptoms?

Response options:
No
Yes, once or twice
Yes, repeatedly

Increase cleaning
Renovation of home premises
Adjusting the ventilation of your home
Disposal or disinfection of household furniture, ozonation
Slept on the balcony
Moved into another apartment

Changing the interior design of the workplace
Avoiding staying in some premises at the place of work/study
Working remotely/studying
Change of job

Avoiding public spaces
Giving up your hobby
Use of vitamins/food supplements or dietary change
Other, what? ______________________________________

15. How useful has it been to you with the information you have received from the following parties in relation to your own indoor air symptoms?

Response options:
Always or almost always useless
Usually useless
Sometimes useful, sometimes not
Usually useful
Always or almost always useful
I haven't received any information from this source.

Occupational physicians
Private physicians
Other doctors

Construction professionals
State, municipal authorities
Research institutes (e.g. National Institute for Health and Welfare, Finnish Institute of Occupational Health, STUK)
Universities

Employers
Professional associations
Patient organizations

Media (television, radio, newspapers)
Social media, discussion forums
Friends and acquaintances

16. Spouse's symptoms or illness related to indoor air
If you don't have a spouse, move on to the next question.

Response options: no *yes, in the last 12 months* *yes, last time more than a year ago*

Has your spouse ever experienced symptoms of indoor air in your home?

Has your spouse ever had symptoms of indoor air at work?

Has your spouse ever undergone medical examinations or treatment for symptoms or illness, which is suspected to be mainly caused by poor indoor air?

Has your spouse ever been on sick leave due to symptoms or illness, which is suspected to be mainly caused by bad indoor air?

17. How many children under the age of 18 do you have?
If you do not have children under the age of 18, please proceed to question 21.

1 2 3 4 5 or more

18. Symptoms or illness related to indoor air in children
Response options: no *yes, in the last 12 months* *yes, last time more than a year ago*

Has any of your children ever experienced symptoms of indoor air in your home?

Has any of your children ever experienced symptoms of indoor air in a school or kindergarten?

Has any of your children ever been examined or treated by a doctor for symptoms or illness, which is suspected to be mainly caused by poor indoor air?

Has one of your children ever been absent from school due to symptoms or illness, which is suspected to be mainly caused by bad indoor air?

19. **Have any of your children been in evasive rooms in the last 12 months because of indoor air problems at a school or kindergarten?**

No
Yes, **how many of your children?**

1 2 3 4 5 or more

20. **Has one of your children been home-schooled in the last 12 months because of indoor air problems at school or kindergarten?**
No
Yes, **how many of your children?**
1 2 3 4 5 or more

21. **Do you think you are more likely to get symptoms than most other people when you're exposed to the following factors?**

Response options:
No
Yes, a little more likely
Yes, much more likely
Yes, very much more likely

Scents
Chemicals
Poor indoor air in buildings
Noise
Electromagnetic radiation
Wind turbines noise

22. **Do the following factors make it difficult for you to do your work, to manage your home affairs or to get along with other people?**

Response options:
None
Quite a bit
Moderately
Quite a lot.
Very much

Scents
Chemicals
Poor indoor air in buildings
Noise
Electromagnetic radiation
Wind turbines noise

LIVING AND WORKING ENVIRONMENT

23. **The type of area you live in?**

City Center, Inner City
Suburb
Rural agglomeration, settlement
Rural sparsely populated area

24. What kind of apartment are you living in right now?

Detached house
Semi-detached or terraced house
High-rise
Somewhere else

25. Do you live in?

Owner-occupied housing (owned by yourself or by a family member)
In a rental apartment
In a partial ownership apartment or right-of-occupancy dwelling
In a service house, rehabilitation home or similar
Somewhere else

26. How would you rate the quality of the indoor air in your apartment?

Very bad
Quite bad.
Neither good nor bad
Quite good
Very good

27. Have the following factors disturbed you in your apartment in the last 4 weeks?

Response options:
None
Yes, sometimes
Yes, every week
Yes, almost every day

Too hot inside
Too cold inside
Draft
Dry air
Stuffy (bad) air
Smell of mould or "smell of ground cellar"
Other unpleasant smell
Dustiness or dirtiness
Insufficient ventilation
Noise
Tobacco smoke

28. Are there in your apartment in the living quarters at this time
Response options:
No
Yes
I don't know

moisture damage
visible mould
Other significant exposures in indoor air

29. Have indoor air problems in your apartment had a debilitating effect on
If there are no indoor air problems in your apartment, please proceed to the next question

Response options:
1 = no
2 = slightly
3 = somewhat
4 = quite a lot
5 = very much

Your human relationships
Your financial situation
Your psychic endurance
Your living arrangements
Your ability to work

30. How would you rate the quality of the indoor air in your place of work or study
If you are not studying or working, please proceed to question 36.

Very bad
Quite bad.
Neither good nor bad
Quite good
Very good

31. How satisfied are you with the management of indoor air issues at your place of work or study?

Very dissatisfied
Quite dissatisfied
Neither satisfied nor dissatisfied
Quite satisfied
Very satisfied

32. Have the following factors disturbed you at work in the last 4 weeks?

Response options:
Not at all
Yes, sometimes
Yes, every week
Yes, almost every day

Too hot inside
Too cold inside
Draft
Dry air
Stuffy (bad) air
Smell of mould or "smell of ground cellar"
Other unpleasant smell
Dustiness or dirtiness
Insufficient ventilation
Noise
Restlessness in the working environment
Tobacco smoke

33. Have indoor air problems in your place of work or study had a debilitating effect on
If there are no indoor air problems at your workplace, please proceed to the next question

Response options:
1 = no
2 = slightly
3 = somewhat
4 = quite a lot
5 = very much

Your relationships with other employees
Your relationships with your manager
Your psychic endurance
Your ability to work
Your work comfort

34. Is your current employer:
State (including universities)
Municipality or joint municipal authority
Private employer
So called third sector (such as associations, foundations, cooperatives or social enterprises)

35. In what type of indoor environment do you mainly work? (select only one option, the most suitable)

School, kindergarten, etc.
Hospital, health centre , etc.
Office-like environment
Production building, mine, construction, etc.
Means of transport, warehouse
Farms
Own home or the home of another
Outdoors
Something else

36. Do you smoke now?

Not at all.
Less than once a month
About 1-2 times a month
About 1-2 times a week
Daily, less than 5 cigarettes a day
Daily, 5-15 cigarettes per day
Daily, more than 15 cigarettes a day

37. Do you smoke yourself or does anyone else smoke indoors regularly in your home?

No
Yes

RISK PERCEPTIONS

38. Assess the extent to which you are currently exposed (you encounter, you are dealing with) to the following factors. Exposure here refers to all exposure, not just harmful exposure.

Response options:
I'm not exposed at all 1
I'm a little exposed 2
I'm somewhat exposed 3
I'm exposed a lot 4
I'm very exposed to 5
I can't say 9

a) Air pollution from transport
b) Wood burning smokes (heating/fireplaces for detached houses)
c) Traffic noise
d) Tobacco smoke (passive smoking, not own smoking)
e) Indoor radon
f) Moisture damage at home
g) Moisture damage in the workplace
h) Moisture damage in public buildings (schools, hospitals, etc.)
i) Other indoor air pollutants (chemicals, etc.)
j) Radiation from mobile phones

39. How much <u>of a risk to your health</u> do you consider <u>your current exposure</u> to the following factors?

Response options:
No risk at all 1
Low risk 2
Moderate risk 3
High risk 4
Very high risk 5
I can't say 9

a) Air pollution from transport
b) Wood burning smokes (heating/fireplaces for detached houses)
c) Traffic noise
d) Tobacco smoke (passive smoking, not own smoking)
e) Indoor radon
f) Moisture damage at home
g) Moisture damage in the workplace
h) Moisture damage in public buildings (schools, hospitals, etc.)
i) Other indoor air pollutants (chemicals, etc.)
j) Radiation from mobile phones

40. And how much <u>risk to human health</u> in general do you consider these factors <u>to be in Finland</u>?

Response options:
No risk at all 1
Low risk 2
Moderate risk 3
High risk 4
Very high risk 5
I can't say 9

a) Air pollution from transport
b) Wood burning smokes (heating/fireplaces for detached houses)
c) Traffic noise
d) Tobacco smoke (passive smoking, not own smoking)
e) Indoor radon
f) Moisture damage at home
g) Moisture damage in the workplace
h) Moisture damage in public buildings (schools, hospitals, etc.)
i) Other indoor air pollutants (chemicals, etc.)
j) Radiation from mobile phones

KNOWLEDGE, TRUST AND ATTITUDES

41. What do you think of the following claims about indoor air problems?

Response options:
Totally disagree 1
Quite disagree 2
Neither agree nor disagree 3
Quite agree 4
Fully agree 5

a) The authorities in my municipality do not take indoor air problems seriously enough
b) My municipality should put more money into dealing with indoor air problems, even if it is out of other services
c) The health damage caused by indoor air problems is downplayed in Finland
d) I have sufficient information on the health risks associated with indoor air
e) I have sufficient knowledge on how to affect indoor air quality in my own home
f) I have sufficient information on how to act when I suspect indoor air problems or have symptoms of indoor air
g) There has been a lot of discussion about indoor air problems in my immediate circle.
h) I am very worried about the health effects of indoor air in Finland

42. What do you think of the following arguments about the health effects of indoor air?

Response options:
Totally disagree 1
Quite disagree 2
Neither agree nor disagree 3
Quite agree 4
Fully agree 5
I can't say 9

a) It is difficult to assess whether a person's respiratory symptoms are caused by indoor air in the building or by something else
b) Symptoms are the best gauge of indoor air quality
c) The assessment of indoor air impurities should be based primarily on the examination of the building and other measurements, rather than symptoms
d) Even minor moisture damage is so harmful to health that it needs to be addressed immediately
e) The presence of actinomyces in the building is a sure sign of the harmfulness of indoor air
f) Most indoor air symptoms are transient
g) Mere concern about indoor air quality can effect symptoms similar to indoor air symptoms
h) Spending time in a moisture-damaged building can lead to indoor air impurities preventing the use of most buildings
i) It is possible to recover from sensitivity to get symptoms from indoor air
j) Buildings can be clearly divided into healthy and problematic buildings
k) Staying in a building, in which one gets symptoms will very likely lead to the development of asthma

43. In your opinion, does a stay in a moisture-damaged building increase the risk of developing the following symptoms or illnesses?

Response options:
Not at all 1
Adds a little 2
Adds somewhat 3
Adds a lot 4
Adds very much 5
I can't say 9

Eye symptoms
Nasal congestion
Dyspnoea
Skin symptoms
Joint symptoms
Fatigue
Headache

Asthma
Allergic rhinitis (e.g. hay fever)
Sinusitis
Other respiratory infections
Rheumatoid arthritis
Cancer
Multiple chemical sensitivity
Electromagnetic sensitivity

44. What do you think of the following arguments about science and research?

Response options:
Totally disagree 1
Quite disagree 2
Neither agree nor disagree 3
Quite agree 4
Fully agree 5

a) Far too little information based on scientific research is used in political decision-making
b) Science cannot be trusted because experts in the same field can completely disagree on something
c) Downplaying science and anti-science has increased in our country recently
d) It is good that research data is challenged on social media and alternative views and explanations are presented
e) There are a lot of untrue allegations in social media and in the public media in general today, which try to contradict scientific claims

45. How much do you trust? Choose the option that describes your opinion on the scale of 'I have no confidence at all – I have complete confidence'.

I have no confidence at all				*I have complete confidence*
1	*2*	*3*	*4*	*5*

public health services
judiciary
State and municipal authorities
Construction industry
Media (television, radio, newspapers)
Social media, discussion forums

46. And how much do you trust these same people in indoor air issues? Choose the option that describes your opinion on the scale of 'I have no confidence at all – I have complete confidence'.

I have no confidence at all				*I have complete confidence*
1	*2*	*3*	*4*	*5*

public health services
judiciary
State and municipal authorities
Construction industry
Media (television, radio, newspapers)
Social media, discussion forums

47. How satisfied are you with the activities of your municipality in indoor air-related matters?

Very dissatisfied
Quite dissatisfied
Neither satisfied nor dissatisfied
Quite satisfied
Very satisfied

48. How satisfied are you with the activities of your municipality, especially in the following matters related to indoor air?

Response options:
Very dissatisfied
Quite dissatisfied
Neither satisfied nor dissatisfied
Quite satisfied
Very satisfied
I don't know.

Quality of maintenance and construction
Solving indoor air problems in schools
Giving information on indoor air matters
Housing inspections carried out by municipal health inspectors

QUALITY OF LIFE

With regard to Questions 49 to 51, we ask you to reflect on your life over the last two weeks.

49. How do you rate your quality of life?

1. Very bad
2. Bad
3. Neither good nor bad
4. For good
5. Very good

50. How satisfied are you with

Response options:
Very dissatisfied 1
Quite dissatisfied 2
Neither satisfied nor dissatisfied 3
Quite satisfied 4
Very satisfied 5

your health?
your ability to cope with your day-to-day activities?
Yourself?
your relationships?
the conditions in your area of residence?

51. Do you have

Response options:
Not at all 1
A little 2
Reasonably 3
Almost enough 4
Quite enough 5

enough energy for your everyday life?
enough money to meet your needs?

52. In the following, we ask you to evaluate your character traits. Read each claim carefully and choose the answer option that best reflects your own opinion.

Response options:
Totally disagree
Quite disagree
Neither agrees nor disagrees
Quite agree
Totally agree

I'm talkative.
I'm careful.
I'm original, I come up with a lot of new ideas.
I easily forgive others.
I worry and grieve easily.
I'm usually quiet and reserved.
I'm often lazy.
I have a vivid imagination.
I'm thoughtful and kind to almost everyone.
I'm relaxed, I can get through stress easily
I'm social and outward-looking
I will persevere in my duties to the end.
I like to think and ponder over things.
Sometimes I'm rude to other people.
I'm tense and I get nervous easily.

I like to lean on to my intuitive impressions.
I can usually count on my intuition.
I often act on my instincts when I make decisions about my activities.
I trust the first impressions I make of people.
I enjoy problems that require thorough reflection
I enjoy intellectual challenges
I prefer complex problems to simple ones
I try to avoid situations that require in-depth reflection on something.

53. A lottery is drawn between the respondents. Do you want to enter the lottery?
Yes
No

54. Response date (dd.mm.yy):

If you wish, you can still write comments below about the topic of the survey.

Thank you for your answers!

Please return this form in the attached fully paid envelope.
