# Supplementary material for: Major changes in indoor air-related symptoms, health worry, and views between 2018 and 2022 in Finland
Source: BMC Public Health. 2025 Oct 10;25:3459. doi: 10.1186/s12889-025-24224-8 (PMC12512946; doi:10.1186/s12889-025-24224-8)
Supplement: Supplementary file 3 — Additional file 3. [file 12889_2025_24224_MOESM3_ESM.pdf]

Supplement to "Major changes in indoor air-related symptoms, health worry, and views between 2018 and 2022 in Finland"

Einar Eidstø[1], Sanna Selinheimo, Kati Huttunen, Vuokko Härmä, Tiina Laatikainen, Pekka Jousilahti, Anniina Salmela, Juha Pekkanen

Corresponding author

Einar Eidstø

einar.eidsto@helsinki.fi

Department of Public Health, Faculty of Medicine, University of Helsinki, Helsinki, Finland

Supplemental Table 1. Differences in the proportional change of prevalence between subgroups from 2018 to 2022

	Sex		Academic degree		Trust in media (TV, radio, etc.) in matters of indoor air		Trust in social media in matters of indoor air		Severity of symptoms		
	Women	*Men*	*Yes*	*No*	*Full trust*	*Little trust*	*Full trust*	*Little trust*	*None*	*Mild*	*Moderate/ severe*
Symptoms at work	-20 %	-29 %	-31 %	-18 %	-46 %	-19 %	-25 %	-24 %	-	-	-
Good self-reported indoor air quality at work/place of study	18 %	6 %	12 %	9 %	17 %	11 %	7 %	13 %	6 %	18 %	25 %
Moisture damage at work is a large health risk	-32 %	-40 %	-41 %	-32 %	-45 %	-35 %	-37 %	-36 %	-38 %	-27 %	-22 %
"I'm very worried about the health effects of indoor air in Finland."	-45 %	-35 %	-51 %	-33 %	-46 %	-41 %	-43 %	-42 %	-42 %	-39 %	-25 %
"Health hazards caused by indoor air problems are downplayed in Finland."	-21 %	-28 %	-24 %	-23 %	-25 %	-24 %	-1 %	-26 %	-26 %	-14 %	-3 %
"Indoor air problems are discussed a lot in my circle of acquaintances."	-38 %	-32 %	-44 %	-28 %	-35 %	-37 %	-40 %	-36 %	-35 %	-32 %	-27 %
"My municipality should spend more money on solving indoor air problems, even at the cost of other services."	-35 %	-27 %	-37 %	-28 %	-20 %	-36 %	-4 %	-34 %	-34 %	-37 %	-3 %
"The officials in my municipality don't take indoor air problems seriously enough."	-29 %	-27 %	-38 %	-20 %	-27 %	-29 %	-7 %	-30 %	-32 %	-18 %	0 %
Disagreement with *"The assessment of indoor air impurities should be based primarily on the examination of the building and other measurements rather than symptoms."*	-25 %	-15 %	-26 %	-21 %	-7 %	-27 %	-12 %	-24 %	-23 %	-24 %	-12 %
Disagreement with *"Even minor moisture damage is so harmful to health that immidiate action has to be taken."*	18 %	28 %	16 %	23 %	-4 %	34 %	-53 %	27 %	20 %	21 %	48 %
Disagreement with *"Mere concern about indoor air quality can effect symptoms similar to indoor air symptoms."*	-17 %	-26 %	-18 %	-21 %	-13 %	-24 %	-9 %	-22 %	-18 %	-35 %	-6 %
Satisfied with municipality's actions relating to "Quality of construction, and maintenance"	53 %	25 %	56 %	27 %	20 %	46 %	13 %	44 %	28 %	88 %	42 %

light grey: p-value<0.1; dark grey: p-value<0.05. P-values for a test of the difference in change between subgroups.
P-values are adjusted for sex, age, and education.
